# Supplementary material for: Evidence for widespread dysregulation of circadian clock progression in human cancer
Source: PeerJ. 2018 Jan 31;6:e4327. doi: 10.7717/peerj.4327 (PMC5797448; doi:10.7717/peerj.4327)
Supplement: Supplemental Information 1 [file peerj-06-4327-s001.pdf]

Supplemental Figures for:

Evidence for widespread dysregulation of circadian clock progression in human cancer

Jarrod Shilts[1], Guanhua Chen[2], Jacob J. Hughey[1,3]

[1] Department of Biomedical Informatics, Vanderbilt University School of Medicine, Nashville, TN, United States
[2] Department of Biostatistics & Medical Informatics, University of Wisconsin-Madison, Madison, WI, United States
[3] Department of Biological Sciences, Vanderbilt University, Nashville, TN, United States

Corresponding author:
Jacob J. Hughey[1,3]

Email address: jakejhughey@gmail.com

Figure S1

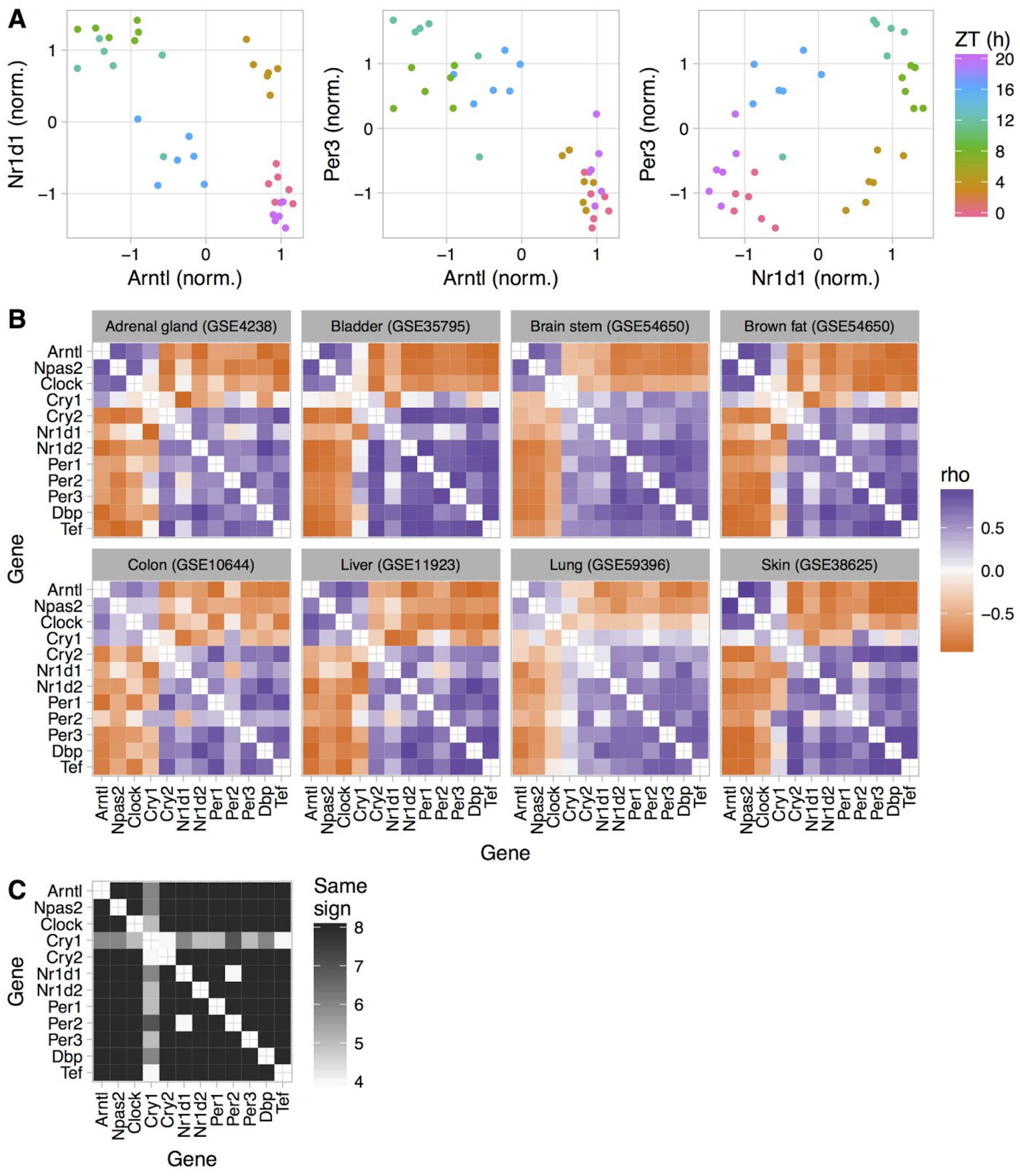

Clock gene co-expression in healthy, wild-type mouse organs. **(A)** Scatterplots of expression for three clock genes in mouse lung (GSE59396). Each point is a sample, and the color indicates zeitgeber time (ZT), where ZT0 corresponds to “lights on.” Expression values of each gene were normalized to have mean zero and standard deviation one. (**B**) Heatmaps of Spearman correlation between each pair of the 12 clock genes in each mouse dataset used to make the reference. Genes are ordered manually by a combination of name and known function in the clock. GSE54650 includes gene expression from 12 organs, but to maintain diversity in datasets, we used data from only two organs. **(C)** Heatmap of consistency in sign of Spearman correlation (across the eight datasets) for each pair of clock genes.

Figure S2

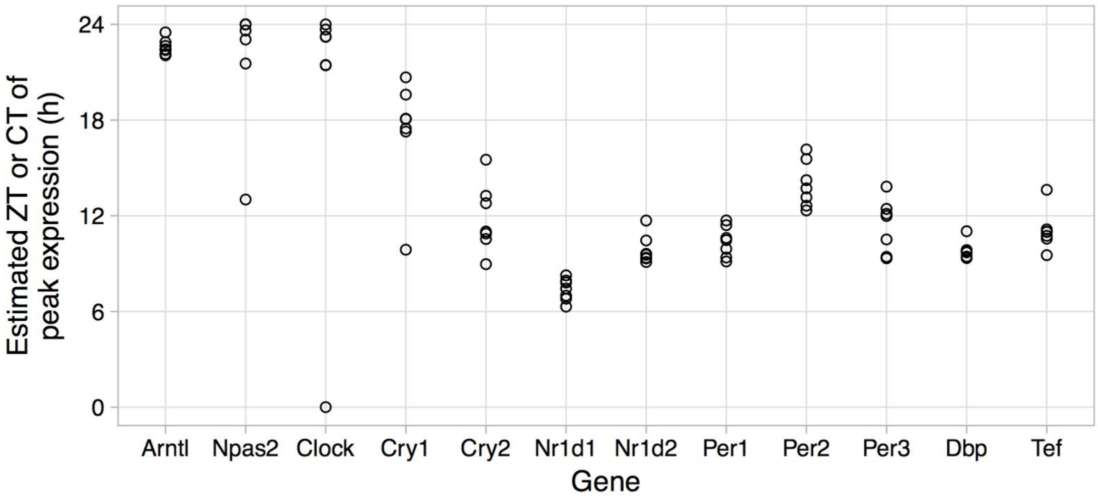

Estimated times of peak expression for the 12 clock genes in each of the eight mouse datasets used to make the reference. Times of peak expression were estimated using ZeitZeiger and the time of day information for each sample, as described in the Methods. ZT refers to zeitgeber time and CT refers to circadian time. ZT0 and ZT24 (or CT0 and CT24, for samples collected under constant darkness) are equivalent. Genes are ordered identically to Fig. 1.

Figure S3

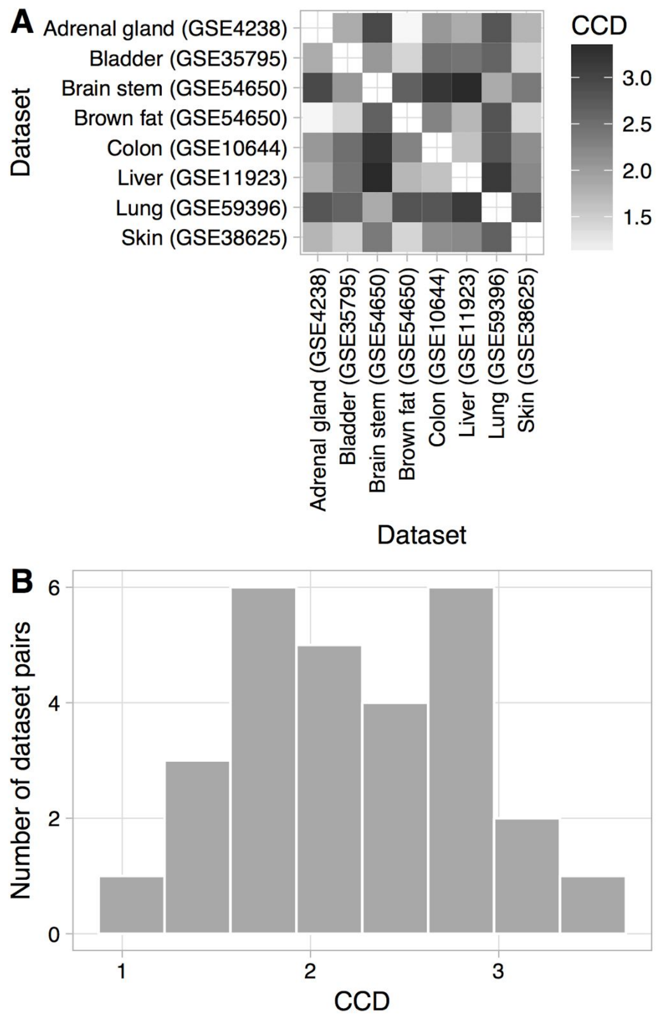

(A) Heatmap and **(B)** histogram of clock correlation distances (CCD) between pairs of datasets used to make the mouse reference. Each unique pair of datasets is only represented once in the histogram.

Figure S4

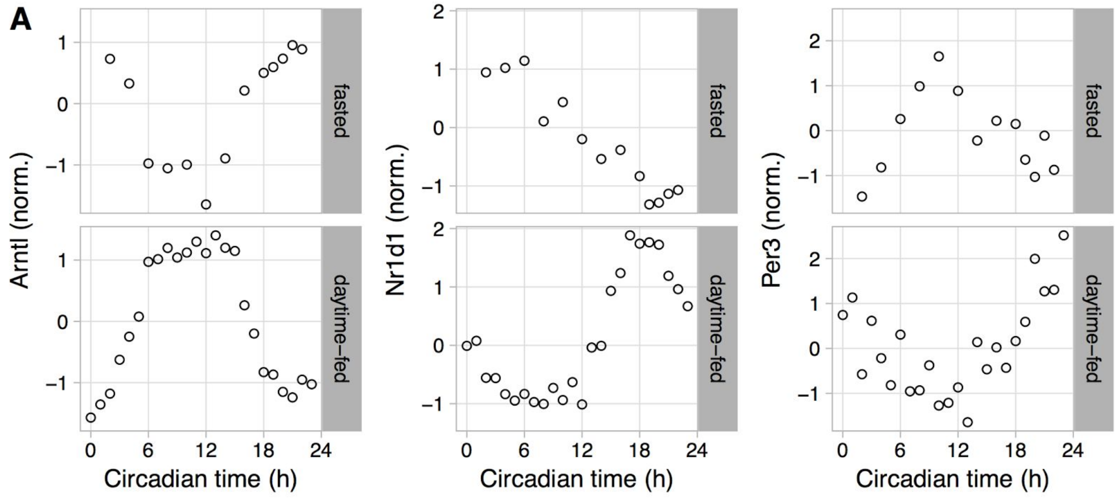

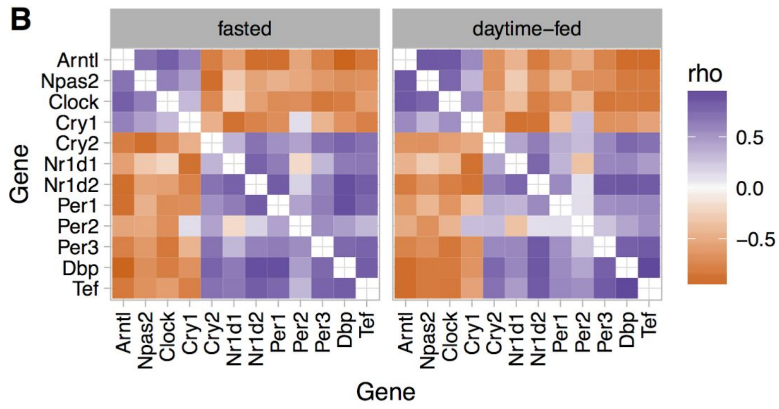

C

Condition	CCD	P−value
daytime−fed	1.81	1.0e−03
fasted	1.71	1.0e−03

Clock gene co-expression is insensitive to phase differences, based on data from liver of wild-type mice (GSE13093). CT refers to circadian time. Daytime feeding (food only available from circadian time CT1 to CT9) shifts the phase of the clock in the liver by 12 h, but does not affect clock gene co-expression. **(A)** Normalized expression of three clock genes over time. **(B)** Heatmaps of Spearman correlation for each condition. **(C)** Clock correlation distance (CCD; relative to the mouse reference) and p-value for each condition (calculated by permutation as described in the Materials and Methods).

Figure S5

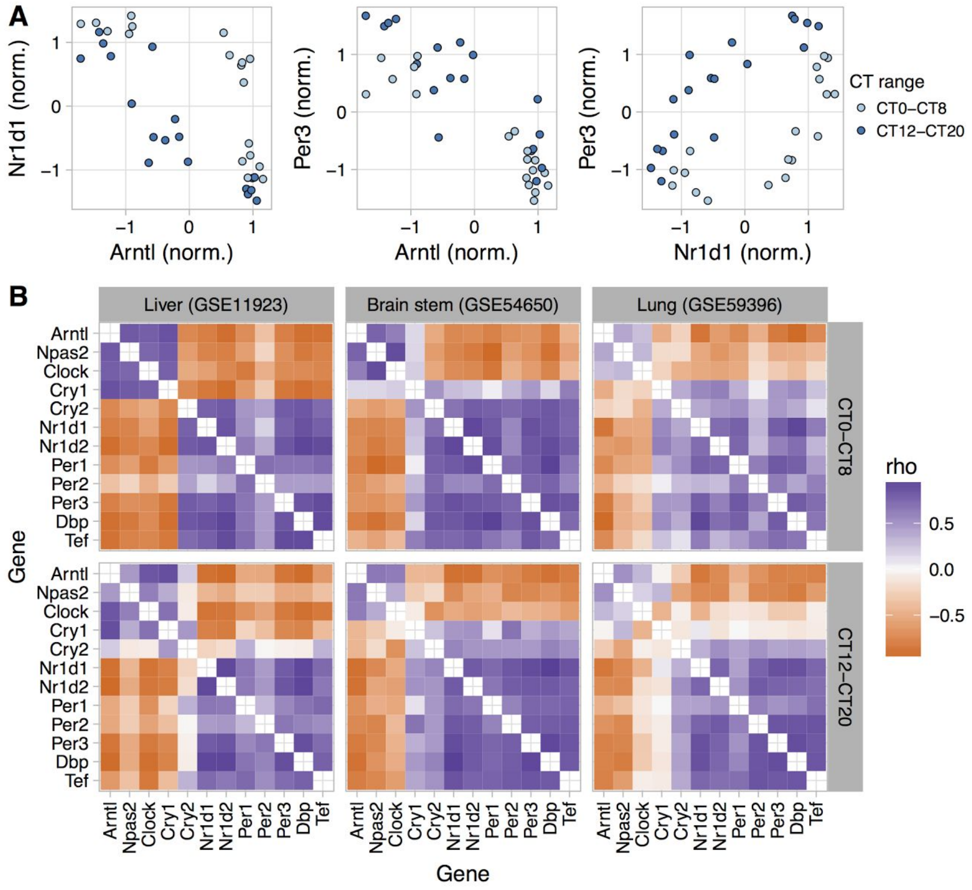

C

Study	CT Range	CCD	P–value
Liver (GSE11923)	CT0–CT8	2.56	1.0e–03
Liver (GSE11923)	CT12–CT20	3.01	1.0e–03
Brain stem (GSE54650)	CT0–CT8	2.37	1.0e–03
Brain stem (GSE54650)	CT12–CT20	2.70	1.0e–03
Lung (GSE59396)	CT0–CT8	3.05	1.0e–03
Lung (GSE59396)	CT12–CT20	2.44	1.0e–03

Clock gene co-expression is robust to incomplete coverage of the 24-h cycle. (**A**) Pairwise scatterplots of expression for three clock genes in mouse lung (GSE59396). Each point is a sample. CT refers to circadian time. Daytime samples are light blue (CT0-CT8), nighttime samples are dark blue (CT12-CT20). Expression values of each gene were normalized to have mean zero and standard deviation one. (**B**) Heatmaps of Spearman correlation for daytime and nighttime samples from three datasets used to make the mouse reference. Samples from GSE11923 and GSE54650 were collected in constant darkness (DD), whereas samples from GSE59396 were collected in alternating light-dark. **(C)** Clock correlation distance (CCD; relative to the mouse reference) and p-value for each dataset and CT range.

Figure S6

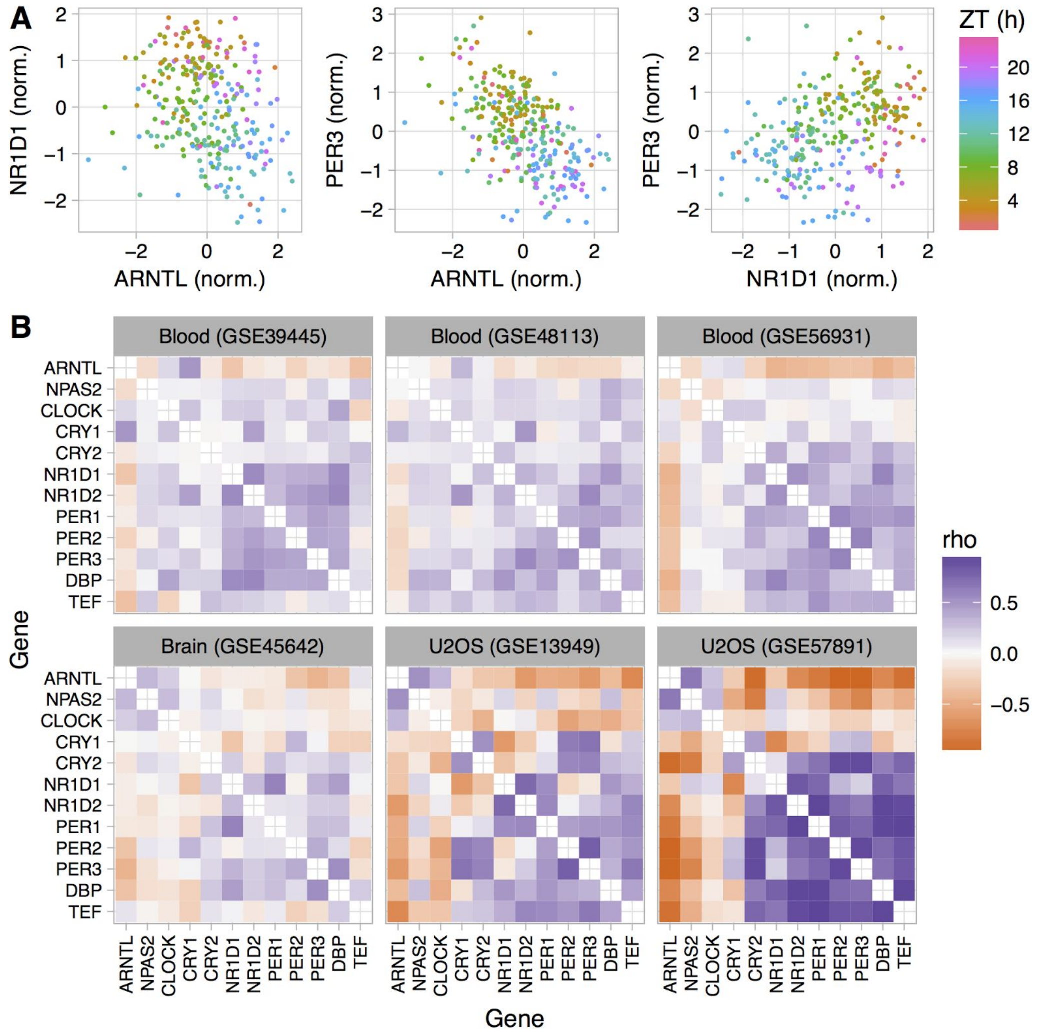

Clock gene co-expression in human datasets that were designed to study circadian rhythms and in which samples are labeled with time of day (or time since synchronization, for in vitro datasets). **(A)** Scatterplots of expression for three clock genes in human brain (GSE71620). For this dataset, gene expression measured in postmortem tissue, with zeitgeber time (ZT) based on time of death. Each point is a sample, and the color corresponds to zeitgeber time, where ZT0 corresponds to sunrise. Expression values of each gene were normalized to have mean zero and standard deviation one. The oscillation that was clear in the mouse data is no longer visible, but the correlations have remained. **(B)** Heatmaps of clock gene co-expression for datasets not shown in Fig. 1D. Datasets from U2OS cells were collected in vitro, the remaining datasets were collected from in vivo tissues.

Figure S7

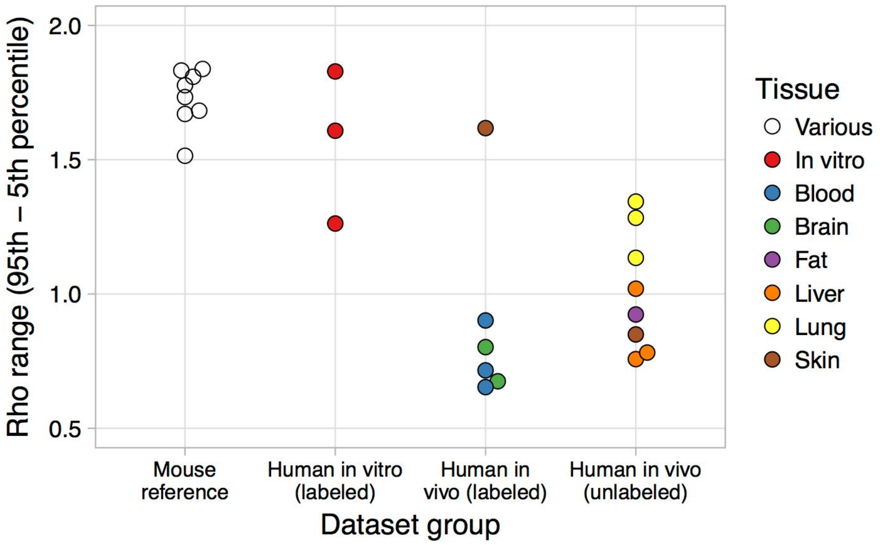

Clock gene co-expression is generally stronger in mouse datasets than in human datasets. In addition, strength of clock gene co-expression in human datasets not labeled with time of day is comparable to that seen in human datasets designed to study circadian rhythms. For each dataset, we quantified the difference between the 95th and 5th percentiles of the distribution of Spearman correlations between pairs of the 12 clock genes (thus, the maximum difference is 2). Each point corresponds to a dataset.

Figure S8

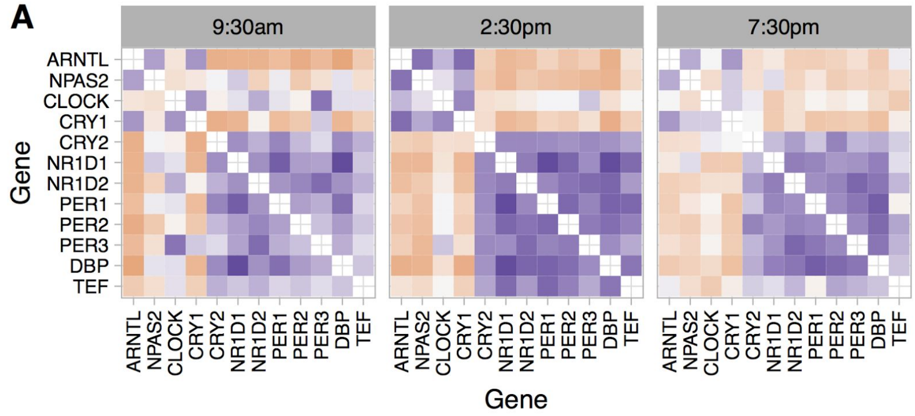

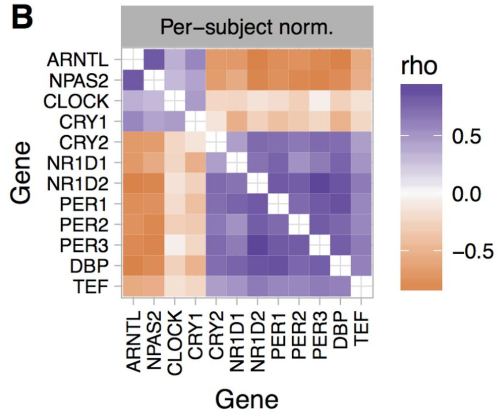

C

Group	CCD	P−value
9:30am	3.97	2.0e−03
2:30pm	2.90	1.0e−03
7:30pm	3.36	1.0e−03
Per−subject norm.	2.03	1.0e−03

Clock gene co-expression in the human skin dataset (GSE35635) is due to co-expression both across individuals at each time-point and across time-points within each individual. Heatmaps of Spearman correlation between each pair of clock genes (**A**) for each of the three time-points and (**B**) after using ComBat to normalize the expression of each gene across individuals. For (B), we only used samples from those individuals for whom samples from all three time-points were collected (18 of 20 individuals). **(C)** Table of clock correlation distance (CCD; relative to mouse reference) and p-values for each time-point and for per-subject normalization.

Figure S9

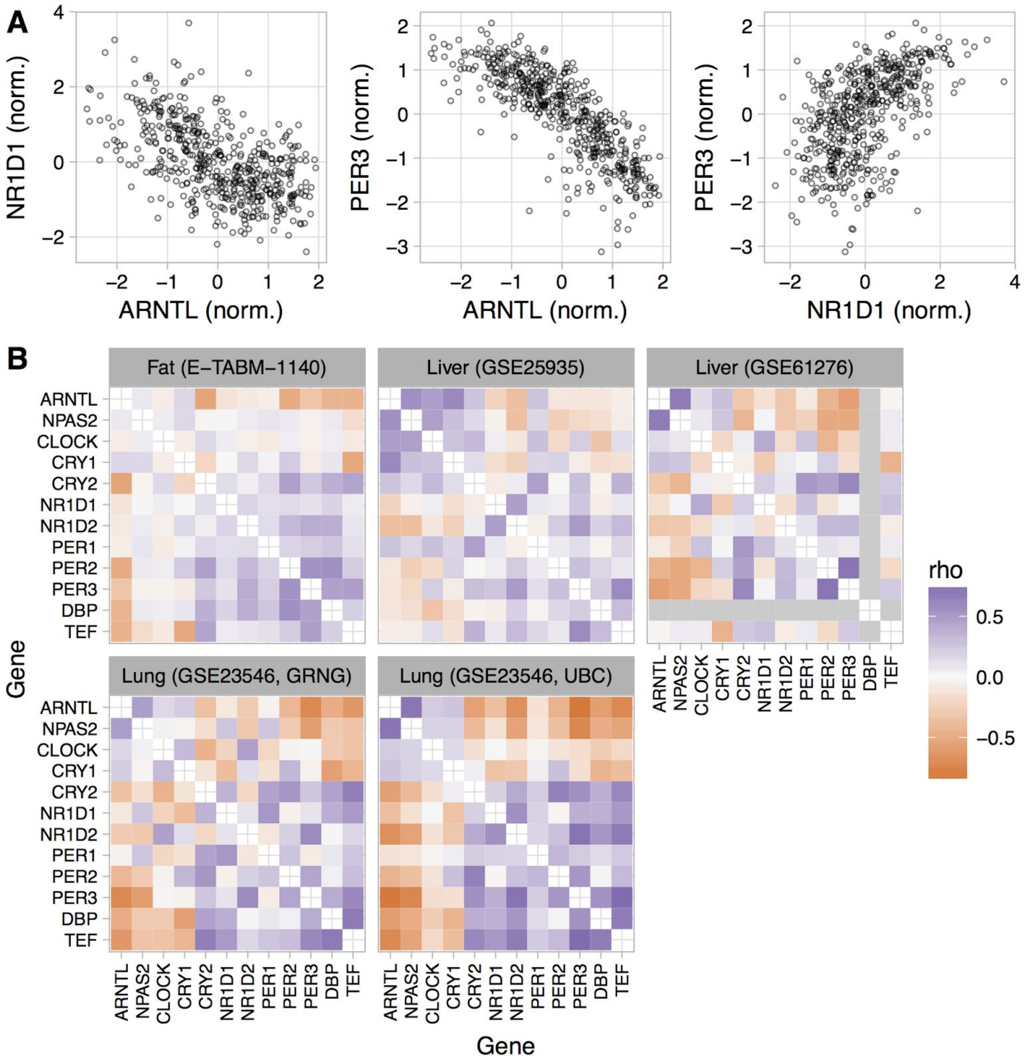

Clock gene co-expression in human datasets that were not designed to study circadian rhythms and in which samples are not labeled with time of day. **(A)** Scatterplots of expression for three clock genes in human lung (GSE23546, Laval). Each point is a sample. Expression values of each gene were normalized to have mean zero and standard deviation one. The oscillation that was clear in the mouse data is no longer visible, but the correlations have remained. **(B)** Heatmaps of clock gene co-expression for datasets not shown in Fig. 1E.

Figure S10

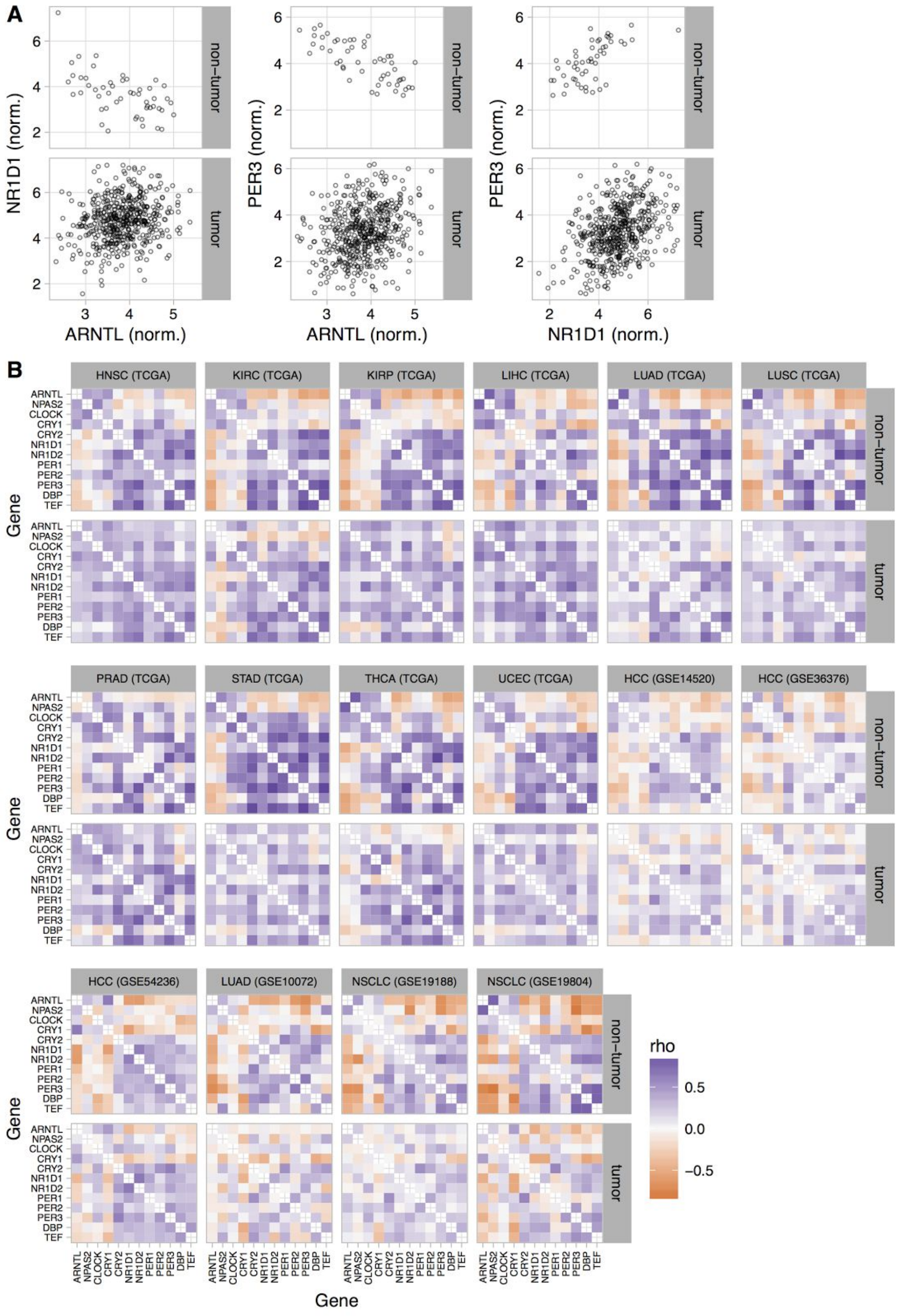
A
NR1D1 (norm.)
ARNTL (norm.)
PER3 (norm.)
ARNTL (norm.)
PER3 (norm.)
NR1D1 (norm.)
non–tumor
tumor
B
HNSC (TCGA)
KIRC (TCGA)
KIRP (TCGA)
LIHC (TCGA)
LUAD (TCGA)
LUSC (TCGA)
PRAD (TCGA)
STAD (TCGA)
THCA (TCGA)
UCEC (TCGA)
HCC (GSE14520)
HCC (GSE36376)
HCC (GSE54236)
LUAD (GSE10072)
NSCLC (GSE19188)
NSCLC (GSE19804)
ARNTL
NPAS2
CLOCK
CRY1
CRY2
NR1D1
NR1D2
PER1
PER2
PER3
DBP
TEF
Gene
rho
0.5
0.0
−0.5

(**A**) Pairwise scatterplots of expression for three clock genes in non-tumor and tumor samples from lung squamous cell carcinoma (LUSC) from The Cancer Genome Atlas. Each point is a sample. Expression values from RNA-seq data are shown in units of $\log_2$(TPM + 1), where TPM is transcripts per million. For ease of visualization, two tumor samples with very high expression of ARNTL are not shown. **(B)** Heatmaps of Spearman correlation between clock genes for non-tumor and tumor samples from human cancer datasets not shown in Fig. 2B. Abbreviations: head and neck squamous cell carcinoma (HNSC), kidney renal papillary cell carcinoma (KIRP), liver hepatocellular carcinoma (LIHC), lung adenocarcinoma (LUAD), prostate adenocarcinoma (PRAD), stomach adenocarcinoma (STAD), thyroid carcinoma (THCA), uterine corpus endometrial carcinoma (UCEC), hepatocellular carcinoma (HCC), non-small cell lung cancer (NSCLC).

Figure S11

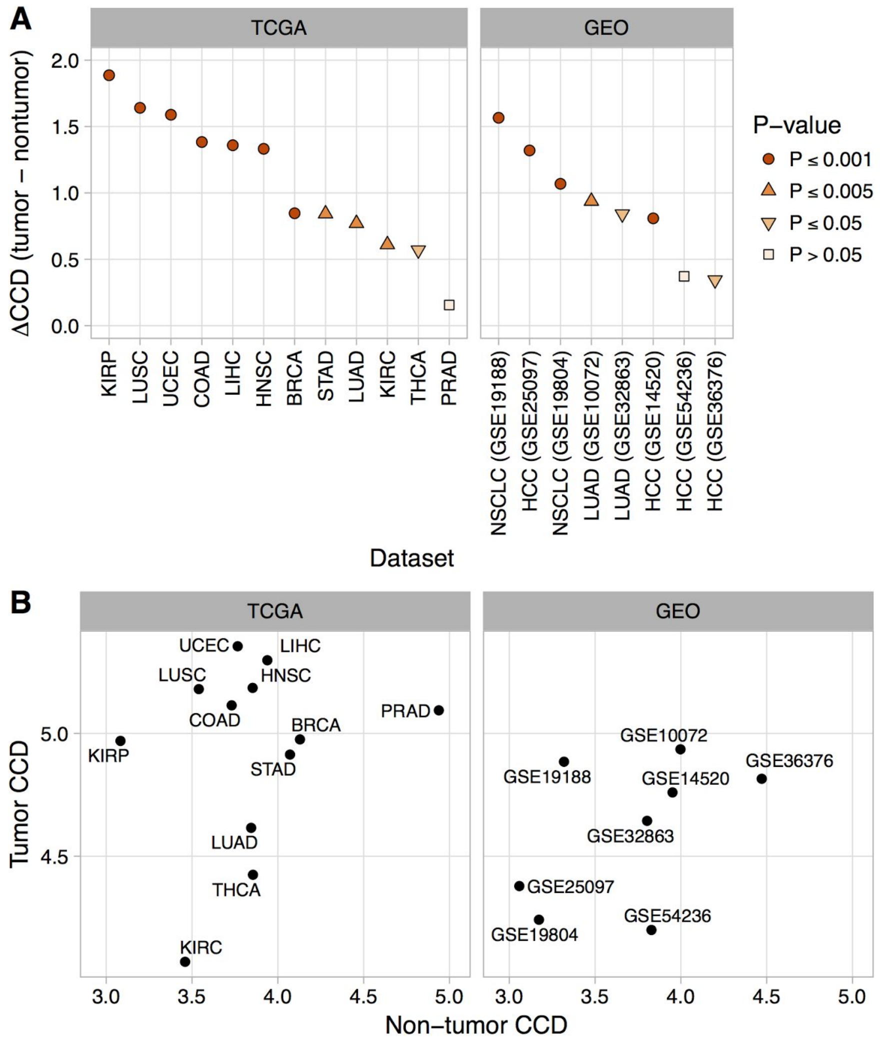

(A) Delta clock correlation distance (ΔCCD) for each human cancer dataset. P-value corresponds to the probability that a random permutation of condition labels could produce a ΔCCD greater than or equal to the one observed. **(B)** Clock correlation distance (CCD) for non-tumor and tumor samples from each human cancer dataset.

Figure S12

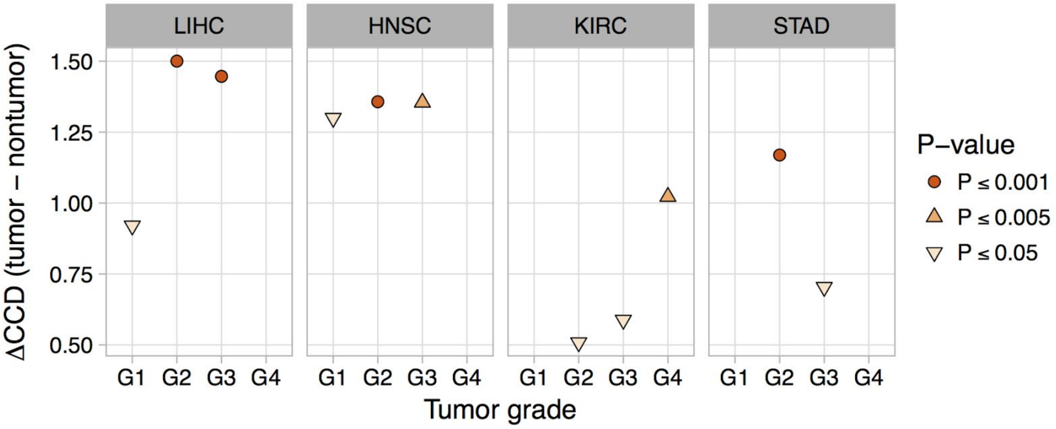

Clock gene co-expression is perturbed in tumors of various histological grades. Plots show delta clock correlation distance (ΔCCD) for all combinations of TGCA cancer type and tumor grade that included at least 50 tumor samples. In each case, ΔCCD was calculated using all non-tumor samples of the respective cancer type. Cancer types are ordered by aggregate ΔCCD.

Figure S13

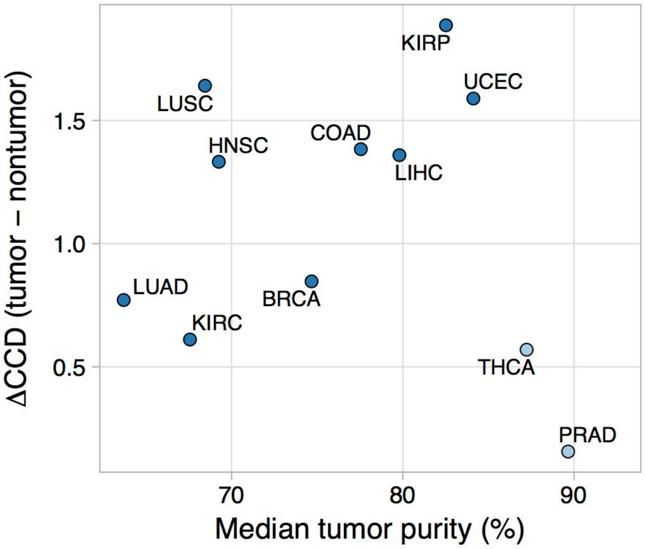

Relationship between tumor purity and delta clock correlation distance (ΔCCD) in TCGA data. Median tumor purity for each cancer type was based on the consensus purity estimate (Aran, Sirota & Butte, 2015). Excluding THCA and PRAD (which are outliers) and STAD (which lacked estimates of tumor purity), the Spearman correlation between median tumor purity and ΔCCD was 0.67 (P = 0.059 by exact test). Abbreviations: head and neck squamous cell carcinoma (HNSC), kidney renal papillary cell carcinoma (KIRP), liver hepatocellular carcinoma (LIHC), lung adenocarcinoma (LUAD), prostate adenocarcinoma (PRAD), stomach adenocarcinoma (STAD), thyroid carcinoma (THCA), uterine corpus endometrial carcinoma (UCEC), hepatocellular carcinoma (HCC), non-small cell lung cancer (NSCLC).

Figure S14

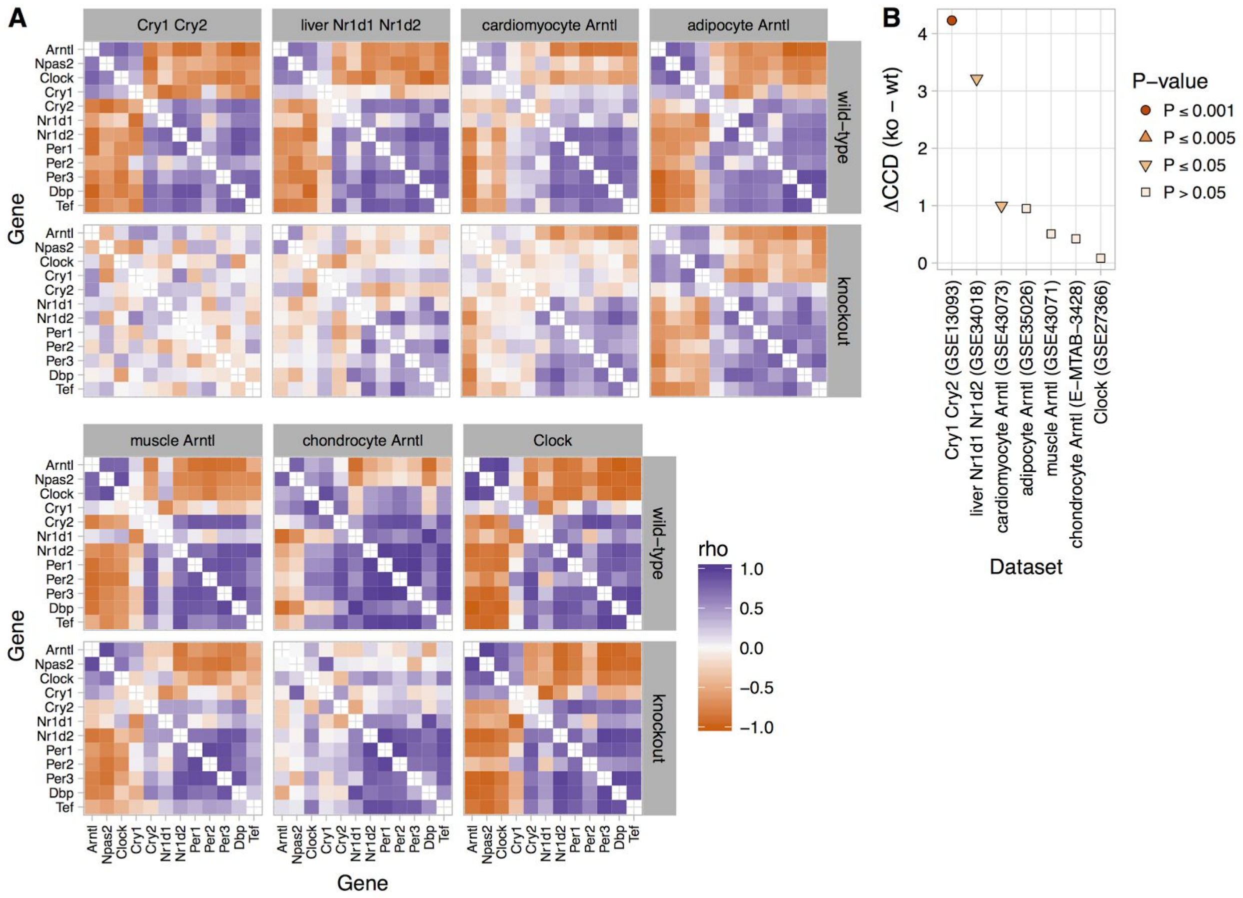

Clock gene co-expression in clock gene knockouts in mice. **(A)** Heatmaps of Spearman correlation between clock genes in wild-type and knockout samples from seven datasets. Knockout samples in each dataset are from mice in which at least one component of the clock was knocked out either in the entire animal or in a specific cell type. Gene expression was measured in various tissues. Datasets are ordered by descending delta clock correlation distance (ΔCCD). For details of datasets, including sample sizes, see Table S1. **(B)** ΔCCD between wild-type and knockout samples in each dataset. Positive ΔCCD indicates that the correlation pattern of the wild-type samples is more similar to the mouse reference than is the correlation pattern of the mutant samples.

Figure S15

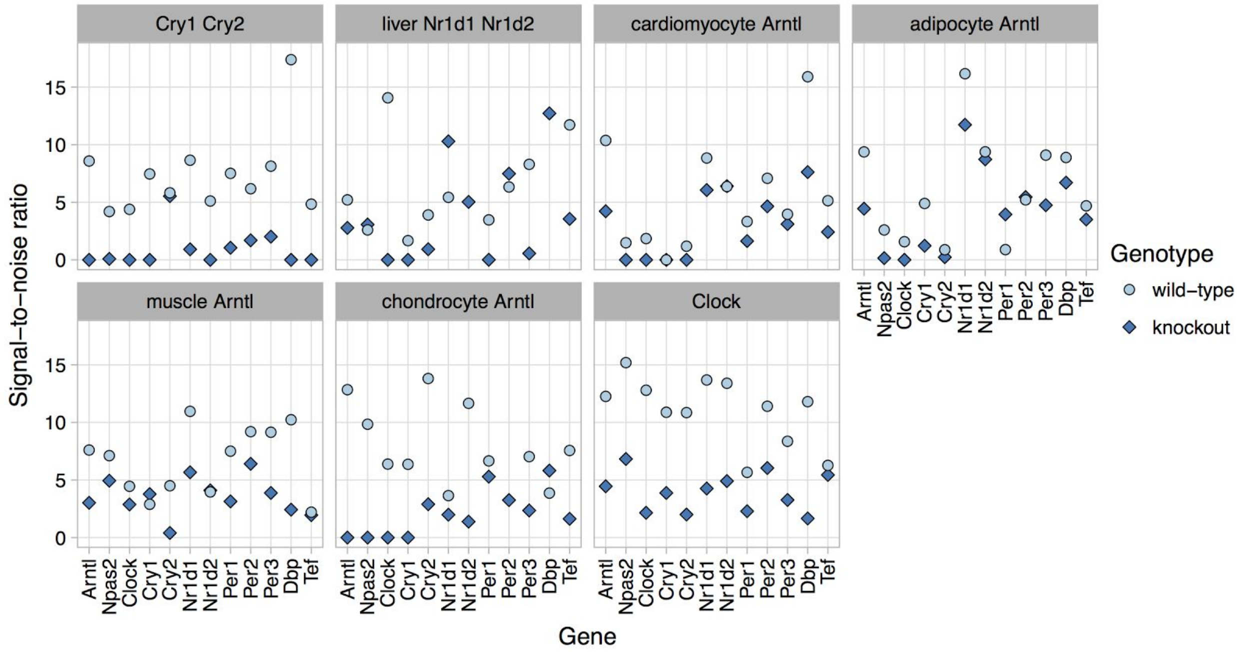

Loss of rhythmicity in clock gene expression in clock gene knockouts. Signal-to-noise ratio (SNR) of circadian expression for each gene in wild-type and knockout samples from each dataset. SNR was calculated using ZeitZeiger (see Materials and Methods for details); the calculation uses each sample's time of day information. For ease of visualization, three outliers corresponding to wild-type samples with SNR>20 are not shown.

References

Aran D., Sirota M., Butte AJ. 2015. Systematic pan-cancer analysis of tumour purity. *Nature communications* 6:8971.
